# The EuResist expert model for customised HAART optimisation: 2010 update and extension to newest compounds

**DOI:** 10.1186/1758-2652-13-S4-O6

**Published:** 2010-11-08

**Authors:** A Pironti, A Sönnerborg, M Zazzi, R Kaiser, D Struck, B Clotet, ÀM Vandamme, F Incardona, T Lengauer, M Rosen-Zvi, M Prosperi

**Affiliations:** 1 Max-Planck-Institut für Informatik, Computational Biology and Applied Algorithmics, Saarbrücken, Germany; 2 Karolinska Institutet, Clinical Virology/Infectious Diseases, Stockholm, Sweden; 3 Università degli Studi di Siena, Molecular Biology Department, Siena, Italy; 4 Universitätsklinikum Köln, Institut für Virologie, Cologne, Germany; 5 Centre de Recherche Public de la Santé, Laboratory of Retrovirology, Luxembourg, Luxembourg; 6 IrsiCaixa, Barcelona, Spain; 7 Katholieke Universiteit Leuven, Clinical and Epidemiological Virology, Leuven, Belgium; 8 Informa S.r.l., Research and Design, Rome, Italy; 9 BM Haifa Research Labs, Machine Learning and Data Mining, Haifa, Israel; 10 Catholic University of the Sacred Heart, Clinic of Infectious Diseases, Rome, Italy

## Background

The design of an optimal highly active antiretroviral therapy (HAART) customised on patient's background and viral genotyping, is still a challenge. EuResist has been the first data-driven system to be implemented as a free web-service for customised HAART optimisation, and was proven to be superior to all existing genotypic interpretation systems (GIS) since it takes into account not only genotype but multiple other variables.

## Data and methods

The EuResist database stores and updates periodically demographic, clinical, and genomic information of HIV+ patients from several countries in Western Europe. The EuResist system is trained on treatment change episodes (TCE) drawn from the EuResist data base, composed of a new drug regimen with a baseline HIV-1 RNA load and a CD4+ count, a baseline viral pol genotype, demographic and previous treatment information. Each TCE is associated to an HIV-1 RNA measurement after 8 weeks, which is used for the definition of virological success (below 500 copies/ml or >2 Log10 reduction from baseline HIV-1 RNA). The system is a combination of three independent machine learning models (based on logistic regression, random forests, and Bayesian networks). The 2010 update has been trained on >5,000 TCE, composed of 20 FDA/EMEA approved nucleoside/tide, non-nucleoside, and protease inhibitors, including the recently approved compounds (RAC) tipranavir, etravirine and darunavir.

## Results

**Figure 1:**
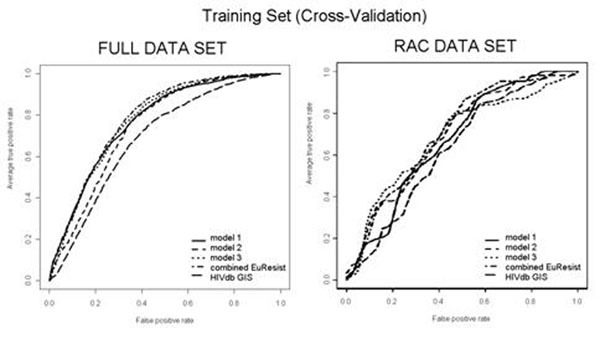


The EuResist combined system performance in predicting the correct virological success of a TCE after 8-weeks (validation set, n=561) exhibited an area under the receiver operating characteristic (AUROC) of 0.8 (whereas Stanford HIVdb GIS assessed to 0.73, p=0.002). The inclusion of therapy history, clinical, and demographic covariates was shown to increase significantly prediction performance. In the subset of regimens containing RAC (n=151), the EuResist AUROC was 0.7 and Stanford HIVdb AUROC was 0.63, not allowing to assess a significant difference owing to the small sample size. See Figure 1.

## Conclusions

Based on patient’s information and virus genotype, the EuResist web-service ranks the most effective HAART regimens by the probability to achieve an undetectable HIV-1 RNA load after 8 weeks. Thus, it might be useful in clinical practice. The 2010 update includes also RAC and achieved fair performance, which is expected to increase with expanding training data.

